# Realizing consumers’ existential dreams via product marketing and mixed reality: a perspective based on affective neuroscience theories

**DOI:** 10.3389/fnins.2023.1256194

**Published:** 2023-09-05

**Authors:** Bin Yin, Yan-Bin Jiang, Jian Chen

**Affiliations:** ^1^Laboratory of Learning and Behavioral Sciences, School of Psychology, Fujian Normal University, Fuzhou, China; ^2^Department of Applied Psychology, School of Psychology, Fujian Normal University, Fuzhou, China; ^3^School of Psychology, Institute of Organizational and Industrial Psychology, Fujian Normal University, Fuzhou, China

**Keywords:** product marketing, mixed reality, affective neuroscience, existential dreams, consumer behavior, mixed reality and affective neuroscience theories

## Abstract

In an era of swift societal changes and escalating consumerism, this paper presents an exploration of an innovative approach that integrates product marketing strategies, mixed reality (MR) technology, and affective neuroscience theories to actualize consumers’ existential dreams. MR, with its unique capacity to blend the virtual and real worlds, can enhance the consumer experience by creating immersive, personalized environments that resonate with consumers’ existential aspirations. Insights from affective neuroscience, specifically the brain’s processing of emotions, guide the development of emotionally engaging marketing strategies, which strengthen the connection between consumers, products, and brands. These integrated strategies not only present a novel blueprint for companies to deepen consumer engagement but also promise more fulfilling and meaningful consumer experiences. Moreover, this approach contributes to societal well-being and prosperity, marking a significant stride in the field of marketing.

## 1. Introduction

### 1.1. Accelerating societal evolution, technological advancement, and the rise of consumerism

In today’s rapidly evolving consumer culture, individuals grapple with an eroding sense of security due to the pervasive nature of the internet and the globalized dissemination of often negatively skewed information, which destabilizes trust in conventional security structures (Gibbs, 2018). To mitigate this anxiety, individuals engage in consumption behaviors, symbolizing their relationships through the exchange of commodities and facilitating a sense of autonomy and control in an increasingly unstable world (Rindfleisch et al., 2009).

Consumption, viewed as a symbolic system, assists individuals in comprehending and articulating self and life, serving several functions including social roles, existential purposes, and transcendent capacities. This transcendent capacity underscores the potential for consumption to exceed the physical significance of objects and material goods (Kurenlahti and Salonen, 2018). As such, consumerism has led to the expression and pursuit of individual identity through diverse consumption patterns, invigorated perpetually by capitalist entities and strategic product marketing (Finkielsztein, 2023). The meteoric rise of technology, with its ability to enhance sensory experiences, has further intensified this cycle of desire. Consequently, the resistance to boredom and the incessant search for fresh stimuli have become increasingly challenging societal norms (Belk et al., 2003).

### 1.2. The influence of product marketing and mixed reality (MR) technology on the actualization of existential aspirations

Corporations employ product marketing as a strategy to evoke consumer emotions, sway decision-making processes, and facilitate an association between brand consumption and societal values (Livas, 2021). Brands may utilize marketing to effectively narrate their stories and promote the values embraced by their consumers. This approach empowers consumers to select products congruent with their self-perception and construct identities through consumption behaviors (van Nuenen, 2016). Propelled by marketing, consumers are enabled to construct self-schemas and aspire to self-meaning through consumption. In other words, consumption possesses a symbolic role, symbolizing our bonds and security within society, and encapsulating certain meanings (Elliott, 1997). Thus, the consumption process simultaneously constitutes a process of self-concept construction. Through this process, consumers can construct the identity they aspire to portray, or alternatively, mitigate the anxiety engendered by the erosion of traditional security sources by forming an association with a brand.

When consumers harbor desires that are challenging to realize within the realm of their actual lives, consumer dreams can offer a balancing mechanism. These dreams provide a fulfilling sensory and emotional experience that aids in the construction and preservation of individual identity (Belk et al., 2003). MR technology, with its capacity to blend real and virtual elements, can facilitate more authentic sensory experiences and unique, diverse stimuli for consumers. It assists corporations with marketing strategies and enriches the shopping experience, fostering deeper connections between brands and consumers (Baker, 2006).

### 1.3. Objectives and research questions

Grounded in the discussions above, this perspective explores the interplay of MR technology, marketing strategies, and affective neuroscience in helping consumers realize their existential dreams. Our research questions are:

1. How does MR technology influence the emotional connection between consumers and products, and its impact on consumer identity construction?
2. How can insights from affective neuroscience and MR application in marketing align with consumer values, assist in the actualization of their existential dreams, and contribute to individual well-being and societal prosperity?

By addressing these questions, we aim to highlight the potential of MR-infused marketing strategies in realizing consumer existential dreams, thereby enhancing consumer experiences and contributing to individual well-being and societal prosperity.

The remainder of this article is structured as follows: We begin by introducing the concept of consumers’ existential dreams and discussing why this is an essential consideration for product marketers. Next, we present the key affective neuroscience frameworks that underpin the application of MR in realizing these dreams. Following this, we illustrate and provide examples of how MR technology is utilized in manifesting these existential aspirations. We conclude by exploring both the individual and societal implications of these phenomena. This progression allows for a comprehensive exploration of the interplay between existential dreams, MR technology, affective neuroscience theories, and consumer behavior, all of which contribute to our unique perspective on this timely and relevant subject matter.

## 2. Existential dreams and consumerism

### 2.1. Definition of existential dreams

Existential anxiety, a fundamental aspect of existentialism, emerges when individuals sense their existence as being jeopardized (Chen and Wang, 2009). This anxiety is predominantly triggered by apprehensions surrounding death, meaninglessness, social relationships, and guilt (van den Bos, 2009). Personal uncertainty constitutes a crucial factor in existential threats and serves as a primary origin of existential meaning (van den Bos, 2009). Personal uncertainty encompasses the subjective feeling of instability concerning one’s values and worldview and the fear of uncontrollability. Scholars investigating existentialism through the lens of intra-individual insecurity have identified three archetypes of insecurity: social concern, concern for meaning, and security (Young et al., 2021). These archetypes encompass individuals’ need for social relationships, the quest for existential meaning, and the attention to achieving relative security within their environment.

It is evident that the theme of individual existence encompasses social relationships, personal development, and the pursuit of life’s meaning. Existentialism may be actualized through consumption, as it enables individuals to establish a sense of belonging and reconstruct self-patterns and identity. Consumption allows individuals to not only exhibit their social status and capabilities through material possessions but also explore life’s meaning in the process (Sweet, 2011; Bauer et al., 2012; Canavan, 2018). Consequently, a consumer’s existential dream should primarily be expressed as an inherent desire for individuals to actualize their potential, seek life’s meaning, and cultivate positive possessions.

### 2.2. The symbolic value of consumption in realizing existential dreams and identity formation

Consumerism, characterized by excess, waste, connectivity, fair trade, and the semiotics of self-formation, enables the realization of the “existential dream” due to the symbolic nature of consumer culture and the distinctiveness that marketing and mass media bestow upon commodities (Meneley, 2018). Once an individual’s basic needs are met, symbolic rather than functional consumption becomes prominent, serving as a tool for restoring self-identity and enhancing the coherence of their self-concept (Yu et al., 2020). Furthermore, it is through consumption that individuals can form relationships with others, bolster self-esteem, and alleviate existential anxiety (Therkelsen and Gram, 2008; Fransen et al., 2011).

Simultaneously, the symbolic value of consumption plays a crucial role in self-construction and identity formation. According to the symbolic consumption theory, consumers can shape and represent their self-concept through consumption, communicating specific messages that alter others’ perceptions of them. This symbolic meaning attributed to a product is co-determined by individual perception and social group understanding (Wang and Chang, 2013). The symbolic value of consumption signifies an individual’s connection and security within society and embodies the process through which they construct their self-identity (Elliott, 1997). For instance, an environmental enthusiast may showcase their identity by choosing sustainable brands, while others can transform themselves by selecting various clothing styles to convey their personality traits or imply their status. Through consumption, people can reveal their true selves or align themselves more closely with their ideal selves. Furthermore, consumption behavior serves as the foundation for constructing identity and status, allowing people to shape their identity, convey the meaning and cultural context behind identity formation, and ultimately interact with others (Ureta, 2007).

## 3. Unraveling affective neuroscience frameworks: the interplay between product marketing, MR and existential needs

### 3.1. The reward system: the catalyst of consumption desire

The human reward system can be triggered by various positive stimuli known as reinforcers. These encompass items such as food, sex, social interactions, and money, all of which can stimulate the mesolimbic dopamine system in the brain. This reward system not only pertains to the anticipation of rewards but also reinforces behaviors that result in these rewards (Berns, 2004; Bayassi-Jakowicka et al., 2021). Remarkably, non-pharmacological activation of the reward system has also been linked to significant pain reduction. For instance, individuals experiencing pain have been shown to have a substantial decrease in their discomfort while viewing images of their romantic partners (Younger et al., 2010).

This mechanism may provide insight into why consumers occasionally seem indifferent to the completion of their purchases, preferring instead to savor the pleasure derived from the shopping process itself (Close and Kukar-Kinney, 2010). Both marketing and MR techniques can stimulate consumers’ reward systems, thus enhancing the enjoyment experienced during consumption. Whether it’s a distinctive product design, engaging brand narrative, or attractive packaging, effective product marketing can, when suitably employed, trigger the reward system. This results in a positive emotional response from consumers. For instance, many consumers purchase a perfume not solely for the scent’s appeal, but also for the narrative behind it. Marketers’ adept storytelling, which evokes pleasant associations, can thus elevate a consumer’s purchasing intention.

In a similar vein, MR technology can incite a sense of novelty and exhilaration, prompting the reward system, and thereby boosting consumer engagement and motivation. Studies have shown that active playing, which allows consumers to experience winning and losing for themselves, is more likely to stimulate striatal reward responses and increase consumers’ active participation in the game compared to vicarious playing such as watching game videos (Kätsyri et al., 2013). For example, MR can visualize gaming scenarios and fuse them with reality, blurring the boundary between the virtual and real world and immersing consumers in the thrill of the game. When consumers are unable to satisfy their material desires, MR can compensate for traditional marketing channels’ limitations, such as static images or videos. Further investigations might explore how the reward system functions in different contexts of virtual or mixed reality, such as educational achievements or shopping experiences, to assess the authenticity and efficacy of MR in simulating real-world experiences. It can offer an alternative form of satisfaction, triggering higher sensory and emotional arousal levels, thereby amplifying the consumer experience (d’Astous and Deschênes, 2005; Yu et al., 2020).

### 3.2. The attachment system: solidifying consumer-brand bonds

The attachment system is another key framework that presupposes the necessity for individuals to forge specific emotional bonds with others—family members, lovers, friends—to facilitate survival and enhance personal well-being (Ainsworth, 1979; Bowlby, 2008). Regulated by neurotransmitters such as oxytocin, dopamine, and serotonin, the attachment system is responsive to social cues like facial expressions, voice tone, and body language (Bai et al., 2009).

In today’s rapidly changing world, where traditional security sources diminish in influence, and the Internet proliferates exposure to negative information, individuals’ existential insecurity escalates. Drawing on Terror Management Theory (TMT), materialistic individuals often assuage their insecurity by strengthening their attachment to brands due to death anxiety (Greenberg et al., 1986; Rindfleisch et al., 2009). This attachment encompasses both the individuals’ personal bond with the brand and their connection to other brand users, fostering meaningful social ties and reducing fear (Dunn and Hoegg, 2014). In a series of three experiments, Reimann et al. (2012) delved into the psychological and neurophysiological mechanisms of how consumers relate to brands. They found that emotional arousal in consumer-brand relationships decreases over time, while the inclusion of the brand into the self increases. For recently formed brand relationships, greater self-reported emotional arousal was observed, while established close brand relationships were associated with decreased emotional arousal and increased inclusion into the self. The study also discovered the moderating role of brand usage frequency, measured skin conductance responses for emotional arousal, and identified the activation of the insula, a brain area related to psychological phenomena such as addiction and interpersonal love, in established close brand relationships. This research enhances understanding of the robust emotional connection between consumers and brands, suggesting that consumers anthropomorphize brands, thus affording them greater empathy and attention (Tuškej et al., 2013; Tuškej and Podnar, 2018). This may help alleviate attachment anxiety and compensate for individual security needs (Proksch et al., 2013). For instance, environmentalists are prone to choose sustainable brands that symbolize social responsibility toward sustainability and environmental protection. Consumers of such a brand are likely to form meaningful social connections and identify with them due to shared environmental beliefs. For individuals experiencing a lack of intimacy, forming connections with a brand can effectively counterbalance their insecurities.

For brands, fostering a healthy connection with consumers is instrumental in enhancing loyalty and brand recognition, thereby encouraging repeat purchases. During the marketing process, it’s essential for brands to understand and integrate the values of their target consumer groups, laying the groundwork for compelling brand storytelling. This alignment amplifies consumers’ identification with the brand, helping them affirm and express their personal identity through it, and nurturing a desire to maintain a positive relationship with the brand (Tuškej and Podnar, 2018). The activation of the attachment system has been shown to play a crucial role in forging a strong emotional connection between brands and consumers. In an innovative approach, some retailers have leveraged Augmented Reality (AR) through mobile applications to promote travel destinations via virtual pet interaction. This strategy increases consumers’ emotional attachment to virtual pets by providing rewarding experiences. Subsequently, the attachment relationship between consumers and these virtual pets can influence their final destination choices (Thirumaran et al., 2021). Studies have also demonstrated that Virtual Reality (VR) can create stimuli to activate specific facets of the attachment system, leading individuals to exhibit attachment behaviors akin to those in real life (Chicchi Giglioli et al., 2017).

MR, offering an even more immersive experience than VR and AR, employs tools like stereoscopic headsets to create highly realistic scenes integrated into the real world. These multi-sensory simulations enhance consumers’ immersive experiences, fostering engaging and pleasant interactions within constructed scenes. This helps boost consumer satisfaction and brand loyalty, illustrating MR’s potential to activate the consumer’s attachment system (Bae et al., 2020). On the other hand, MR opens up exciting opportunities for multi-dimensional narratives, enabling brands to craft more engaging and interactive multi-sensory stories. These vivid tales can facilitate increased brand-consumer interaction, ignite positive emotions, and amplify consumers’ emotional investment. This heightened connection not only improves consumers’ perceptions of brands but also aids in establishing a more stable and enduring relationship. As an example, MR can create positive associations with the shopping experience itself, shaping consumers’ attitudes and willingness to buy in meaningful ways (Nakevska et al., 2012; de Regt et al., 2021).

### 3.3. Self-determination theory: guiding consumer motivation and satisfaction

Self-determination theory, the final framework, posits that individuals inherently seek autonomy, competence, and relatedness (Deci and Ryan, 2000). These psychological needs are foundational to shaping their motivation and overall well-being: Autonomy emphasizes control over personal decisions and actions; competence refers to the aspiration for mastery and skill development; relatedness encapsulates the desire for social connections and a sense of belonging. Meeting these needs can heighten the intrinsic motivation and “stickiness” of an individual within virtual environments (Huang et al., 2019). For example, within an immersive learning setting, students can autonomously navigate and construct knowledge. Virtual environments, in this context, can ignite students’ motivation and help sustain their focus (Chao et al., 2021).

In the similar vein, the deployment of MR technology can aid individuals in becoming more autonomous, i.e., more cognizant of their preferences during decision-making, boost their confidence and comfort in decision-making, and elevate consumer satisfaction with their shopping experience by diminishing shopping uncertainty (Baker, 2006; Barba et al., 2012). Furthermore, MR can provide individuals with an amplified sense of autonomous control and assist in liberating and expressing their perceptions, beliefs, and attitudes toward stimuli and even themselves within the physical world, enabling consumers to construct and manage a virtual or ideal identity (Schnack et al., 2020). The brand choice during consumption is also a reflection of the consumer’s values and social capabilities, and individuals can display social masks through their consumption behaviors by autonomously selecting the aspects of themselves they wish to exhibit (Coelho et al., 2018). The symbolism of commodities can also assist in restoring a consumer’s threatened self-perception if they feel their self-image is somehow compromised (Saenger et al., 2020).

During the consumption process, individuals inevitably engage in social interactions. Research indicates that virtual environments’ gaming features provide users the chance to fulfill their psychological needs for autonomy, competence, and relatedness. When these needs are met, users show increased intrinsic motivation, particularly in virtual settings where they feel empowered and autonomous (Huang et al., 2019). In a marketing context, these needs can be fulfilled and consumers’ intrinsic motivation and satisfaction can be elevated through strategies like product customization, consumer feedback, and social recognition. Likewise, social feedback and sharing can foster a sense of community and closeness. For instance, anime fans, unlike cultural tourists whose primary objective is learning and growth, often visit anime meccas driven by personal sentiments and the pursuit of social belonging. Such snap tourism also reflects their motivation to engage with fellow fans, and MR can facilitate an enhanced blend of reality and fantasy for them (Kirillova et al., 2019).

## 4. Navigating reality and fantasy: MR’s potential to augment consumer experiences

### 4.1. Expanding boundaries: the intriguing features of MR

MR resides at the intersection of the physical and virtual domains, embodying a distinct synthesis that surpasses the individual capacities of both VR and AR. Where VR immerses users within an entirely virtual environment, MR carefully melds the real and virtual, maintaining a tangible connection to the physical surroundings. On the other hand, unlike AR’s superficial overlay of digital data on the real world, MR’s virtual aspect provides sensory stimuli and reacts to user movements, affording an immersive experience that transcends what AR can accomplish (Kaplan et al., 2021). MR elevates AR’s foundational concept by forging a seamless, bi-directional interaction between real and virtual realms, leveraging artificial intelligence-generated imagery, sound, and tactile feedback to create an unparalleled connection (Nair and Patel, 2018). It amalgamates the immersive nature of VR with the situational relevance of AR, granting users the ability to realistically engage with objects within a fluid universe of virtuality and reality (Pala et al., 2022). This integration culminates in a multifaceted, real-time encounter that surpasses the constraints of VR’s detachment from reality and AR’s mere digital augmentation (Kaplan et al., 2021).

The ramifications of MR’s capabilities extend far beyond technological novelty. By adroitly overcoming the inherent limitations in both VR and AR, MR enables users to immerse themselves more profoundly within the simulated reality. Through facilitating real-time interactions between the virtual and actual worlds, MR charts a course that transcends traditional barriers, guiding users in intertwining the strands of imagination with the tangible reality. MR represents not merely an evolution but a revolution in experiential technology, erasing the distinctions between the real and the virtual, and inaugurating a novel epoch of interaction and sensory exploration (Speicher et al., 2019; Nasr and El-Deeb, 2023).

### 4.2. Revolutionizing consumer experience through MR: from brand interactions to innovative marketing strategies

MR technology bridges the gap between consumer desire and reality. When conventional marketing channels fall short, MR can provide an immersive sensory and emotional experience, thereby personalizing the customer journey and promoting identity construction (d’Astous and Deschênes, 2005). On one hand, through MR’s advanced sensory simulations, consumers can engage with products in a manner that is both vivid and tangible, which can cultivate enhanced consumption experiences and bolsters purchase intentions (Cesari et al., 2021; Jin et al., 2021). On the other hand, MR can elevate consumer-brand interactions, generating dynamic emotional connections that resemble human relationships and enable engaging brand storytelling (Alcañiz et al., 2019). Just as meaningful interactions in a rich game storyline can influence players’ emotional attitudes and behaviors, brands can use MR to create meaningful interactive narratives that evoke consumers’ emotional and cognitive responses, subtly convey brand concepts and values to consumers, and encourage consumers to think or identify (Rosenberg, 2023). The MR-enabled interactivity fosters a closer relationship between consumers and retailers, facilitating a better understanding of products and more informed purchasing decisions (Schmid and Huber, 2019; Li et al., 2022; Barta et al., 2023).

MR’s success is evident across a myriad of fields and industries (Table 1). For instance, in the beauty industry, MR empowers patients to visualize and plan surgical outcomes, enhancing satisfaction (Nair and Patel, 2018). This technology has also revitalized the fashion sector, enabling virtual try-ons (Jin et al., 2021), while industries like automotive, travel, and tourism have harnessed MR for product exploration and virtual tours in relevant contexts, enriching consumer experiences. The multidimensional immersion facilitated by MR augments consumers’ sense of presence and engagement, fostering a more profound understanding of brand concepts and narrative experiences akin to real life (Meenar and Kitson, 2020). As a continuum between the real and virtual, MR mirrors the physical environment more closely than AR and VR, enhancing realistic social interactions and emotional resonance (Dasgupta et al., 2018; Moustafa and Steed, 2018). This multifaceted interaction aligns perfectly with contemporary consumer needs, paving the way for pioneering marketing strategies, such as virtual makeup try-ons in e-commerce platforms, and interactive museum displays (Hammady et al., 2020; Sung et al., 2021). In particular, some museums have leveraged Microsoft’s Hololens to intertwine historical interactive visualizations with tangible artifacts, supplementing or even supplanting traditional tour guide. This allows visitors to engage directly with valuable cultural relics, enriching their experience, prolonging their stay, and potentially enhancing the museum’s profitability.

**Table 1:** Application of MR in realizing consumers’ existential dreams in various industries.

Industry	Application of MR	Impact on consumer experience	References
Arts, museums & cultural heritages	Interactive displays of virtual humans, items and surroundings	Enriched visitor experiences and long-term benefits	Bäck et al. (2019), Bae et al. (2020), Hammady et al. (2020), Barrile et al. (2022), Komianos (2022), Sylaiou and Fidas (2022), and Trunfio et al. (2022)
Automotive	Product exploration, virtual test drives	Improved decision-making in design, production and customer engagement	Borsci et al. (2015) and Varga et al. (2020)
Beauty	Visualization of surgical/makeup outcomes	Enhanced planning and satisfaction	Nair and Patel (2018), Mangtani et al. (2020), and Yun and Hwang (2023)
E-Commerce	Interacting with virtual form of products located in targeted real places	Improved purchasing experience and confidence	Jain and Werth 2019; Fu’adi et al. (2021), Kowalczuk et al. (2021), and Baltierra (2023)
Education	Immersive and interactive learning experiences	Enriched educational engagement and outcomes	Bäck et al. (2019), Maas and Hughes (2020), Tang et al. (2020), Shaytura et al. (2021), Yannier et al. (2022), and Aguayo and Eames (2023)
Fashion	Virtual try-on of clothes in real environment	Informed purchasing, increased excitement	Jin et al. (2021), Silva and Bonetti (2021), and Silvestri (2022)
Healthcare	Enhanced realism in medical training and treatment procedures	Improved effectiveness and patient care	Fu et al. (2022), Goharinejad et al. (2022), Palumbo (2022), Sahija (2022), and Sivananthan et al. (2022)
Retail	Interactive storytelling, presentation of ideas without physical limitations	Stronger connection, faster and more accurate consumer interaction, positive emotions	Cavazza et al. (2004), Dehghani et al. (2020), and Sung et al. (2021, 2022)
Travel & tourism	Create highly realistic scenes integrated into the real world, activation of multiple senses for immersive experiences	Enhanced customer satisfaction, increased enjoyment, increased brand loyalty	Kirillova et al. (2019), Thirumaran et al. (2021), Abass and Zohry (2022), Buhalis and Karatay (2022), and Mkwizu (2023)

In retail, MR transcends the traditional physical environment, giving rise to immersive worlds wherein narratives can be crafted and controlled. This powerful persuasive tool infuses the consumer experience with meaning, while also breaking the boundaries of physical space to present ideas, improve engagement, and evoke positive emotions (Cavazza et al., 2004; Dehghani et al., 2020). Case in point, a café using MR to allow interaction with historic sweatshirts elicited overwhelmingly positive customer feedback, illustrating the technology’s potential in brand promotion (Cheng and Furusawa, 2018). Additionally, the travel industry is employing MR to elevate travel experiences, engaging multiple senses to immerse travelers in their journeys, thereby enhancing enjoyment and brand loyalty (Bae et al., 2020). In all these facets, MR revolutionizes consumer experience, transforming brand interactions into innovative, immersive marketing strategies.

### 4.3. Overcoming barriers: challenges and future prospects of MR technology

Despite its immense potential in acting as a conduit between brands and consumers, nurturing enriched communication and stronger emotional connections, MR technology still poses several obstacles. These obstacles include challenges such as correctly interpreting the physical world, GPS accuracy, substantial investment cost, and varied acceptance across different consumer demographics (Scholz and Smith, 2016; Rositi et al., 2021). Microsoft’s HoloLens 2, for instance, marked an improvement over its predecessor, delivering enhanced hardware and software, improved comfort, and stability. However, obstacles persist, including a limited field of view, unsuitable weight for prolonged wear, and insufficient battery life—factors that hinder its widespread adoption (Palumbo, 2022). Other prevalent issues such as 3D motion sickness, low accessibility, and excessive virtual experience loading times not only disrupt immersion but may also cause physiological discomforts like disorientation and nausea, dampening consumer satisfaction (Cometti et al., 2018; Pala et al., 2022). The seamless fusion of high-quality content with reality is vital for the success of MR in marketing, but these barriers must be addressed first.

The ongoing evolution of MR technology, coupled with advancements in artificial intelligence, bodes well for transformative shifts in marketing. Future exploration in the realm of MR technology might begin with further enhancing the quality of immersive experiences, tailoring them to align with consumers’ lifestyle aspirations and existential dreams. This could encompass virtual endeavors that allow individuals to visualize and interact with personalized fashion, travel, or home environments. Researchers might delve into the potential of MR for fostering social interaction within retail spaces, creating virtual communities or shared shopping experiences that resonate with shared interests and values. The feasibility of multi-dimensional immersion that assimilates all senses can transform fields such as culinary arts or perfumery, providing consumers with holistic sensory engagement. In relation to sales and product design, the interactive pursuit of MR technologies can instigate improved discourse between brands and consumers, intensifying brand identification and solidifying emotional connections. Furthermore, the spatial immersion inherent to MR may stimulate consumer engagement and responses in intricate environments, enabling customers to scrutinize product functionality in various contexts and interpret the narratives brands aspire to convey. The refinement of AI, crucial to augmenting this immersion, must progress in conjunction with MR’s integration into marketing, with the concept of spatial computing serving as a prime illustration (Delmerico et al., 2022; Egliston and Carter, 2022). Ethical considerations and accessibility should be prioritized, ensuring that MR technology resonates with diverse demographics. Integration with existing retail environments may enhance in-store experiences, and realizing existential dreams through lifelike simulations could present new horizons for education and training. Ultimately, MR promises to offer an advertising approach that is more participatory, authentic, and attention-grabbing compared to traditional modes, holding the potential to overhaul traditional marketing practices, heighten experiential consumption, and unlock a new era of empathetic and dream-realizing consumer engagement.

## 5. Final thoughts: steering the course of MR-infused product marketing - a neuroscientific approach

**Figure 1:**
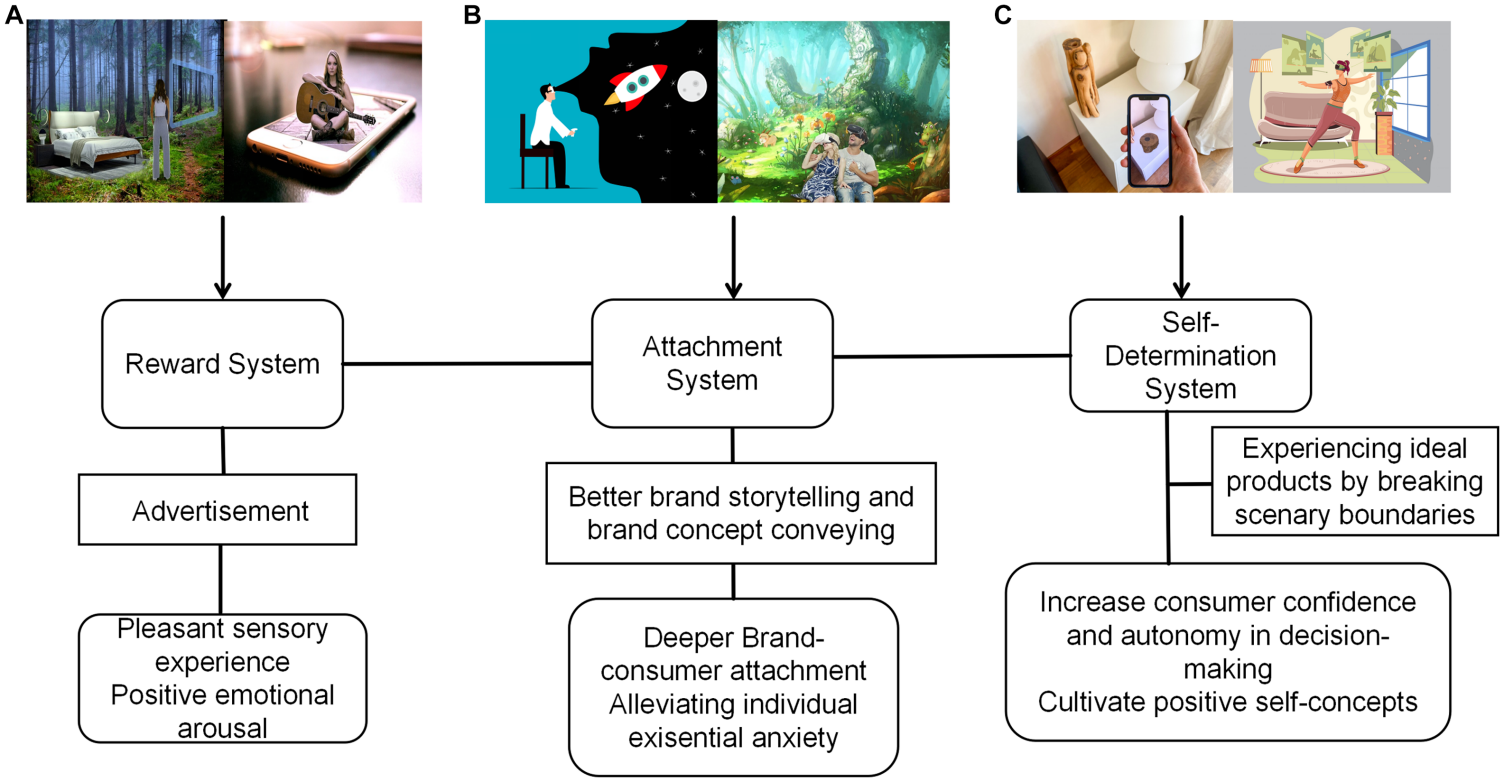
Mixed reality technology’s advancement of marketing and realization of consumer existential dreams based on affective neuroscience theories. **(A)** Utilizing the reward system: this segment illustrates how mixed reality technology is employed in advertising to display product features and benefits, maximizing consumers’ delightful sensory experiences. By merging virtual reality and interactive elements of the real environment, the technology allows for an intuitive understanding of a product’s function and performance. Examples include creating specific atmospheres in MR advertisements to simulate physical sensations, or engaging consumers’ multisensory system with auditory accompaniments. The resultant positive sensory experiences enhance interest, attention, and emotional bonds between consumers and brands. **(B)** Leveraging the attachment system: this segment emphasizes MR’s capability as an influential tool for brand storytelling. Through virtual reality, brands can vivaciously present core concepts and values, heightening consumer immersion and creating a firsthand brand story experience. Such storytelling enhances emotional warmth, fosters deeper emotional connections, and aids in existential exploration. Whether it’s understanding the brand’s spirit of continuous innovation or seeking domestic warmth, this interactive approach boosts brand identity, loyalty, and diminishes existential fears. **(C)** Emphasizing self-determination theory: here, MR’s potential is portrayed in transcending spatial constraints, enabling at-home real product experiences. Through simulating real-world contexts and employing MR devices, consumers can explore product characteristics and usage scenarios, increasing confidence and autonomy in purchasing decisions. Examples include personalized product customization and concrete function experiences through, such as non-paused exercise guidance. These interactions augment consumers’ skills, self-confidence, control over life, and realization of existential dreams. Images within the diagram are sourced from Pixabay.com (Non-commercial use permitted) and Unsplash.com (Non-commercial use permitted).

The intersection of MR technology and artificial intelligence gives rise to an innovative, immersive, and dynamic marketing approach. The combination of these technologies with product marketing creates a potent tool for understanding and catering to consumers’ needs, enabling existential aspirations to be actualized. Moreover, insights from affective neuroscience, particularly those relating to the reward system, attachment system, and self-determination theory, provide valuable blueprints for guiding the future of MR-infused product marketing (Figure 1).

Rooted in the understanding of the human reward system, MR can stimulate consumers’ reward systems, enhancing the enjoyment experienced during the consumption process. By carefully designing MR experiences that align with the positive stimuli that trigger the reward system, such as novelty, exhilaration, and immersive engagement, brands can heighten consumer engagement and motivation, consequently bolstering brand loyalty and satisfaction. The attachment system framework offers a perspective on strengthening consumer-brand bonds. Given the escalating existential insecurity in the digital age, brands can exploit MR to foster deeper, more impactful connections with consumers. By integrating brand personality and values into immersive and interactive MR environments, brands can cultivate trust, familiarity, and loyalty. MR can help brands align more closely with the values of their consumer base, fortifying the emotional bond between consumers and brands, and enhancing brand recognition and repurchase behavior. Further, the self-determination theory identifies autonomy, competence, and relatedness as core psychological needs shaping consumer motivation and overall well-being. Through MR technology, these needs can be satisfied, offering consumers more control and autonomy over their shopping experiences, enhancing their sense of competence and mastery, and fostering a sense of relatedness and belonging. For instance, MR can provide personalized and customizable shopping experiences, amplifying consumers’ sense of autonomous control and satisfaction.

Additionally, the successful integration of MR technologies in marketing strategies can make substantial contributions to both individual and societal well-being. At the individual level, MR can facilitate personal satisfaction and identity formation through brand association, allowing consumers to express their values, desires, and beliefs. From a societal viewpoint, MR-powered marketing strategies can guide consumption trends toward more sustainable practices. Brands that align their values with social responsibility can attract socially conscious consumers, influencing societal norms, and fostering collective well-being. However, it’s vital to consider potential challenges such as high development costs, the digital divide, and diverse consumer acceptance. Collaborative efforts among neuroscientists, marketers, and technology developers will be vital to navigate these obstacles.

In conclusion, the integration of MR technology with product marketing, backed by insights from affective neuroscience, presents a promising pathway to realize consumers’ existential aspirations. The potential to apply MR technology in harmony with affective neuroscience’s understanding of human behavior opens up exciting new vistas for the future of marketing. Continued research and cross-disciplinary collaboration will be essential in harnessing the transformative potential of this approach, with mixed reality serving as a pivotal component in this new era of consumer-brand interactions.

## Data availability statement

The original contributions presented in the study are included in the article/supplementary material, further inquiries can be directed to the corresponding authors.

## Author contributions

BY: Conceptualization, Funding acquisition, Investigation, Resources, Supervision, Validation, Writing – original draft, Writing – review & editing. Y-BJ: Investigation, Visualization, Writing – original draft. JC: Conceptualization, Funding acquisition, Supervision, Validation, Writing – review & editing.

## Funding

The author(s) declare financial support was received for the research, authorship, and/or publication of this article.

This work was supported by the general project of National Social Science Foundation for Education in China, *Research on the Influence Mechanism and Intervention of Youth’s Sense of Existence*, Project No. BBA200038.

## Conflict of interest

The authors declare that the research was conducted in the absence of any commercial or financial relationships that could be construed as a potential conflict of interest.

## Publisher’s note

All claims expressed in this article are solely those of the authors and do not necessarily represent those of their affiliated organizations, or those of the publisher, the editors and the reviewers. Any product that may be evaluated in this article, or claim that may be made by its manufacturer, is not guaranteed or endorsed by the publisher.
